# The ZEB1 Transcription Factor Is a Novel Repressor of Adiposity in Female Mice

**DOI:** 10.1371/journal.pone.0008460

**Published:** 2009-12-24

**Authors:** Jessica N. Saykally, Soner Dogan, Margot P. Cleary, Michel M. Sanders

**Affiliations:** 1 Department of Biochemistry, Molecular Biology and Biophysics, University of Minnesota, Minneapolis, Minnesota, United States of America; 2 The Hormel Institute, University of Minnesota, Austin, Minnesota, United States of America; University of Las Palmas de Gran Canaria, Spain

## Abstract

**Background:**

Four genome-wide association studies mapped an “obesity” gene to human chromosome 10p11–12. As the zinc finger E-box binding homeobox 1 (ZEB1) transcription factor is encoded by the *TCF8* gene located in that region, and as it influences the differentiation of various mesodermal lineages, we hypothesized that ZEB1 might also modulate adiposity. The goal of these studies was to test that hypothesis in mice.

**Methodology/Principal Findings:**

To ascertain whether fat accumulation affects ZEB1 expression, female C57BL/6 mice were fed a regular chow diet (RCD) ad libitum or a 25% calorie-restricted diet from 2.5 to 18.3 months of age. ZEB1 mRNA levels in parametrial fat were six to ten times higher in the obese mice. To determine directly whether ZEB1 affects adiposity, wild type (WT) mice and mice heterozygous for *TCF8* (*TCF8*+/−) were fed an RCD or a high-fat diet (HFD) (60% calories from fat). By two months of age on an HFD and three months on an RCD, *TCF8+/−* mice were heavier than WT controls, which was attributed by Echo MRI to increased fat mass (at three months on an HFD: 0.517±0.081 total fat/lean mass versus 0.313±0.036; at three months on an RCD: 0.175±0.013 versus 0.124±0.012). No differences were observed in food uptake or physical activity, suggesting that the genotypes differ in some aspect of their metabolic activity. ZEB1 expression also increases during adipogenesis in cell culture.

**Conclusion/Significance:**

These results show for the first time that the ZEB1 transcription factor regulates the accumulation of adipose tissue. Furthermore, they corroborate the genome-wide association studies that mapped an “obesity” gene at chromosome 10p11–12.

## Introduction

Obesity and its related metabolic disorders have become an international health concern, especially because of their alarming increase in the young [1]. Excessive white adipose tissue puts individuals at risk for medical conditions such as Type II diabetes, cardiovascular disease, and cancer. Therefore, considerable interest exists in defining the molecular pathways that regulate the development of adipocytes and their ability to store lipids in hopes of elucidating new preventative measures and treatment options.

Adipocytes primarily derive from multipotent mesenchymal stem cells (MSCs) that reside in bone marrow and the stroma of adipose tissue [2]. The conversion of MSCs to preadipocytes activates an extensive transcriptional cascade that regulates terminal differentiation (for recent reviews, see [3], [4]). While several transcription factors such as PPARγ (nuclear peroxisome proliferator-activated receptor gamma) and the C/EBPα family are key components of this cascade, other modulatory transcription factors continue to be discovered [5]. This report identifies one such transcription factor, the ZEB1 (zinc finger E-box binding homeobox 1) transcription factor.

As much as 70% of obesity can be attributed to polygenetic traits [6], and numerous genome-wide linkage studies have attempted to define loci that segregate with a predisposition to obesity [6]–[10]. To increase the likelihood that these association scans are detecting genetic rather than environmental conditions, they are often done on children. Four studies [9], [11]–[13], two using children and young adolescents, are of particular interest as they found linkage in a region of chromosome 10 (10p11–12) that harbors *TCF8*, the gene that encodes the ZEB1 transcription factor. So far, no viable candidate obesity/anti-obesity gene in this region has emerged. We hypothesize that mutations or polymorphisms in *TCF8* contribute to childhood/adolescent obesity.

A number of lines of correlative evidence support the hypothesis that ZEB1 plays a role in adipogenesis and/or lipogenesis. Expression of ZEB1 is high in mesenchymal tissues, including adipose tissue [14]. Moreover, ZEB1 modulates the differentiation of the myogenic, osteogenic, and chondrogenic mesenchymal lineages [15]–[19] and can even direct which lineage differentiates from the multipotent C2C12 mesenchymal cell line [20]. Thus, it is reasonable to propose that ZEB1 can also affect the differentiation of the adipogenic lineage. Additionally, ZEB1 mRNA expression increases following stem cell differentiation into adipocytes in culture [2], and it is higher in obese women compared to those of normal weight [21], although nothing has been done as yet to investigate either of those observations. The goal of our studies was to determine whether ZEB1 affects adipose accumulation in mice.

ZEB1 (also called δEF1, TCF8, Nil-2-a, AREB6) is a large transcription factor of 1117 amino acids in humans that is conserved from worm [22], [23] to man (for review, see [19]). It binds to DNA via two zinc finger clusters at its N- and C-termini, and it recognizes target genes through a modified E-box sequence (5′-CACCT(G)). Mechanistically, little is known about how ZEB1 regulates gene expression, but it can either repress or activate target genes [24], [25]. The regulation of ZEB1 is also understudied. We were the first to report that it is induced by estrogen [24], but it is also induced by progesterone [26], suggesting that ZEB1 plays an important role in female physiology. Interestingly, high expression of ZEB1 becomes independent of estrogen in endometrial and ovarian carcinomas [27]. Other regulators of ZEB1 include NF-kB and the TGF-β family [19]. ZEB1 is also induced by p63 in neurons in response to ischemia, and ZEB1 appears to serve protective role in the central nervous system [28]. Because *TCF8* null mice are perinatal lethal [25], even less is known about its target genes or normal physiological roles, especially in the adult. ZEB1 is predominantly known for its ability to modulate epithelial to mesenchymal transitions (EMT) in development and cancer by downregulating E-cadherin and other genes encoding cell adhesion and basement membrane proteins [29], [30]. While the effects of ZEB1 on EMT have been studied extensively, its role in the development and differentiation of tissues is complex and less well understood (for review, see [19]). However, ZEB1 is at the intersection of multiple mesenchymal developmental pathways [15]–[19], raising the possibility that it also affects adipogenesis.

The experiments in this study were undertaken to directly investigate whether or not ZEB1 modulates adipogenesis and/or lipogenesis *in vivo* using female mice, which were chosen because estrogen induces ZEB1 [24], [26], [27], [31]. As considerable data indicate that estrogen protects against weight gain [32], [33], it seems plausible that ZEB1 might mediate estrogen's effects on adiposity. The results with wild type (WT) mice demonstrate that ZEB1 mRNA levels in adipose tissue increase dramatically with weight gain and plateau as the rate of weight gain decreases. To directly test whether ZEB1 affects this weight gain, mice heterozygous for *TCF8* (*TCF8*+/−) were fed a regular chow or high fat chow diet for up to 5 months, and the effects on body weight, fat mass, activity level, food intake, and glucose processing were determined. The resulting data indicate that haploinsufficiency of ZEB1 is sufficient to elicit significant fat accumulation, regardless of diet. Furthermore, in cell culture models of adipogenesis, ZEB1 expression increases with differentiation and lipid accumulation, as it does in the mice. These data are the first to show that ZEB1 plays a protective role against obesity. They are also the first to support the genome-wide association studies that mapped a human ‘obesity’ gene to chromosome 10p11–12 [9], [11]–[13].

## Materials and Methods

### Animals and Genotyping

All experiments were done in accordance with the University of Minnesota Research Subjects Protection Program in adherence with federal, state, and local regulations. The IACUC protocol number is 0609A91913. All mice were maintained in a University of Minnesota specific pathogen-free animal facility, where light and temperature were regulated. *TCF8* null mice were generated by Dr. Yujiro Higashi and colleagues (Osaka University, Osaka, Japan) by β–galactosidase (β-gal) insertion into Exon 1 as described previously [15], [25], and heterozygous (*TCF8*+/−) males were provided by Dr. Jennifer Richer, University of Colorado, Aurora, CO. The colony was generated and maintained on a C57BL/6 background. Female breeder mice were purchased from Charles River Biological Laboratories (Wilmington, MA). Genomic DNA was isolated from ear snips, and polymerase chain reactions (PCR) were performed to determine genotype. To generate products, Choice-Taq (#CB4050-2 Denville Scientific, Metuchen, NJ) with its accompanying 10X buffer and 25 mM dNTPs were used. PCR reactions were done for 35 cycles consisting of 95°C for 30 sec, 61°C for 30 sec, and 72°C for 1 min. Products were amplified using the allele-specific primers designed by Jennifer Richer (University of Colorado) listed below. The *TCF8* forward and reverse primers were used to detect wild type (WT) animals (193 bp product), and the *TCF8* forward and β-gal reverse primers were used to detect heterozygous animals (537 bp product).

*TCF8* Forward: 5′-AGCACTATTCTCCGCTACTCCAC-3′

*TCF8* Reverse: 5′-ACCGCACCTGGTTTACGACACTC -3′

β-gal Reverse: 5′-AACCGTGCATCTGCCAGTTTGAG-3′

### Animal Diet and Body Composition

**Figure 1:**
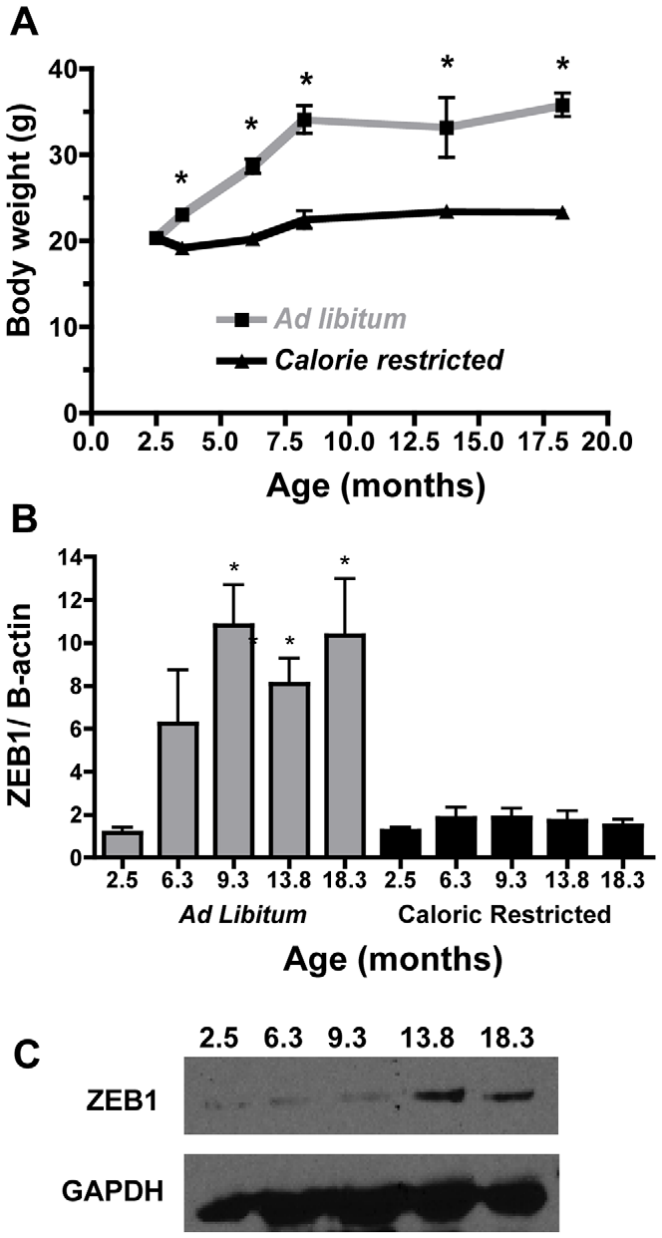
ZEB1 mRNA expression increases concomitantly with weight in WT female mice. Mice were fed regular chow *ad libitum* or a diet restricted to 75% of the calories of the *ad libitum* group (calorie restricted). (**A**) Body weights (g) were recorded as indicated for mice that were fed *ad libitum* (gray line) or calorie restricted (black line). n = 3–7 mice/group. (**B**) Corresponding ZEB1 mRNA expression in parametrial fat was determined by quantitative SYBR real time PCR. ZEB1 mRNA was expressed relative to β-actin mRNA, *ad libitum* (gray bars), calorie restricted (black bars). n = 3–7 mice/group (**C**) Western blot confirming that ZEB1 protein expression increases in response to increased body weight in mice fed *ad libitum*. GAPDH was used as a loading control. Individual lanes are labeled as months of age.

The caloric-restricted mice used for Figure 1 were treated as described previously [34]. Briefly, C57BL/6 female mice 10 weeks of age were fed a purified regular chow diet (RCD) (AIN-93M, Harlan Teklad, Madison, WI) *ad libitum* or were fed a restricted amount of a diet modified to provide 75% of the calories consumed by the *ad libitum* group [35]. Thus, only the absolute amount of carbohydrate differed between the two dietary regimens. Body weights of the mice were recorded weekly until sacrifice at specific time points as indicated, with the longest being 18.3 months of age. Parametrial fat was harvested at the indicated times (Figure 1), snap frozen in liquid nitrogen, and stored at −80 °C.

For the remaining experiments, female *TCF8*+/− and WT controls were placed for the indicated times on either a RCD (#2018, Harlan Teklad Global Diets, Indianapolis, IN) or a high fat diet (HFD, 60% of calories from fat, #F3282, Bio-Serv, Frenchtown, NJ) following weaning at 21 days of age. *TCF8+/−* and WT mice were age matched with 8–12 mice per group. Body weights were recorded once a week from 5 weeks of age until sacrifice. Mice were sacrificed at 2, 3, 4, and 5 months for tissue collection; consequently, as the experiment progressed, fewer mice were available to record body weights. Fat pads were harvested and weighed, snap frozen in liquid nitrogen, and stored at −80°C. Whole body composition was determined by use of nuclear magnetic resonance imaging (MRI) technology produced by Echo Medical Systems LTD (Houston, TX). Mice were placed in a tube and analyzed on the accumulation 4 setting, which is specific for mice, to determine total fat mass, lean mass, body fluid, and water weight.

### Real-Time PCR (qPCR) Analysis

Total RNA was harvested from the parametrial fat of female mice using the RNeasy Lipid Tissue Mini Kit, (Qiagen #74804, Germany). cDNA synthesis was performed using 2 µg total RNA as the template, oligo(dT)_13_ (Integrated DNA Technologies, Coralville, IA), and AMV reverse transcriptase (Roche #10109118001, Germany). ZEB1 mRNA was measured on a BioRad iCycler (#170-8740, Hercules, CA) using iQ SYBR Green Supermix (BioRad #1708885) and normalized to β-actin mRNA. Cycling was performed per the manufacturer's instructions with annealing temperatures of 61°C and 61.8°C for ZEB1 and β-actin, respectively. Primers used to amplify were as follows:

ZEB1 Forward: 5′-CGAGTCAGATGCAGAAAATGAGCAA-3′

ZEB1 Reverse: 5′-ACCCAGACTGCGTCACATGTCTT-3′

β-actin Forward: 5′-CAAAAGCCACCCCCACTCCTAAGA-3′

β-actin Reverse: 5′-GCCCTGGCTGCCTCAACACCTC-3′

### Protein Expression

ZEB1 protein levels were measured by western immunoblotting. One hundred mg of parametrial adipose tissue was homogenized in detergent-free lysis buffer with Mini Complete Protease Inhibitor (Roche, Indianapolis, Indiana). To remove lipid, samples were spun at 10,000×g for 15 min at 4°C, and the floating lipid removed. The remaining sample was pipetted into a clean tube and incubated on ice for 30 min with lysis buffer that contained detergents. Samples were spun at 10,000×g for 15 min at 4°C, and the protein in the supernatant quantified using the Bio-Rad *DC* assay. Thirty mg of protein was electrophoresed on a 6% polyacrylamide gel and transferred to PVDF membrane. Immunoblotting was done using α-ZEB1 antibody at a 1:200 dilution (#H-102, Santa Cruz, Santa Cruz, CA) and α-GAPDH antibody at a1:1000 dilution (# MAB 374 Chemicon, Billerica, MA). Secondary anti-mouse (Santa Cruz) and anti-rabbit (Santa Cruz) peroxidase antibodies were used, respectively. Proteins were detected using Pierce Super Signal West Pico Chemiluminescent Substrate (#34080 Rockford, IL).

### Glucose Tolerance Tests

Age-matched mice were fasted for 6 hr prior to testing. The blood glucose baseline was determined using Roche ACCU-CHEK Aviva (Germany, #04528280001) blood glucose monitor and strips. Mice were injected with an intraperitoneal bolus of glucose dissolved in 0.9% saline. The amount of glucose injected (0.5–3 mg/kg) was dependent upon body weight and is indicated in the figure legend. Blood glucose was monitored every 15 min for the first hr and every 30 min for the second hr or until the glucose levels returned to baseline levels.

### Food Intake

Mice were housed individually following weaning at 21 days. Caloric intake was measured at 2.5 months of age. Cages were adapted with a wire mesh raised 1/8 inch off the bottom, allowing food crumbs to fall through for collection and weighing. One hundred g of regular chow was placed in each cage and after 3 days the remaining food and crumbs were weighed, and food consumption was calculated.

### Activity Measurements

The activities of WT and *TCF8+/−* mice were determined as described previously [36]. Briefly, 3-month-old mice were housed singly in mock chambers to acclimatize to their surroundings for 24 h before being placed in chambers that record both horizontal and vertical movement based on laser breaks. Prior and during the experiment, mice were fed regular chow.

### Cell Culture

3T3-L1 cells were obtained from ATCC and maintained in DMEM with 10% calf serum and 1% penicillin/streptomycin. To differentiate these preadipocytes into mature adipocytes, the cells were grown to confluency, and 48 hours later they were treated with differentiation media consisting of DMEM with 10% fetal bovine serum supplemented with bovine insulin, dexamethasone, and isobutylmethylxanthine [37]–[39]. Two days following treatment, the medium was refreshed with DMEM +10% FBS and 10% bovine insulin.

C3H10T1/2 cells were differentiated as in [40]. In brief, cells were maintained in DMEM with 10% fetal bovine serum and 1% penicillin/ streptomycin. Fifty ng/mL of BMP-4 (bone morphogenetic protein-4) (R& D systems, Minneapolis, MN) was added to the cells when they reached ∼75% confluency. Once cells were confluent, they were treated to differentiate with the same cocktail as the 3T3-L1 cells.

### Statistics

**Figure 2:**
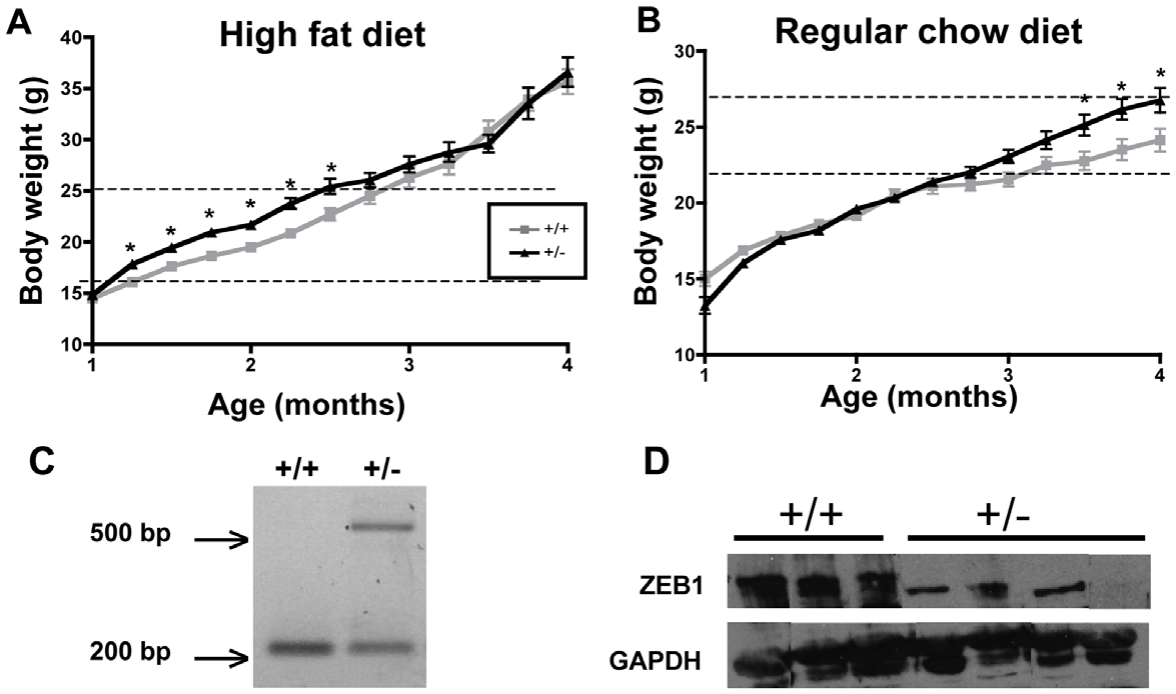
Female mice missing one *TCF8* allele gain weight more readily. Mice were weaned to (**A**) a diet high in fat (60%) or (**B**) regular chow diet, and body weights (g) of female *TCF8 +/−* (black line) or WT (gray line) mice were recorded weekly as indicated. n = 8–34 mice per group, with the number of mice decreasing due to sacrifices at 2 and 3 months. (**A**) Significance calculated by Student's *t*-test between age-matched groups, using the Bonferroni post-test correction set at p<0.005. (**B**) Significance calculated by Student *t*-test between age-matched groups. All mice from 12–18 weeks have p<0.05. However, when corrected for Bonferroni's post-test at p = 0.003 only those from 14–16 weeks are significant. Significance is denoted by *. (**C**) An example of the genotyping that was done to identify *TCF8+/−* and WT mice. The band at ∼500 bp is from the β-galactosidase gene, which was inserted in one of the *TCF8* alleles. The band at ∼200 bp represents *TCF8*. (**D**) ZEB1 protein levels in parametrial adipose tissue of WT (n = 3) and *TCF8+/−* (n = 4) female mice at 3 months of age. GAPDH was used as a loading control.

Data were analyzed using the statistics package GraphPad Prism version 4 (San Diego, CA). Data are represented as means +/− standard error of the means (SEM). Unless otherwise noted, Student's *t*-test was used to compare two groups. Two-way analysis of variance (ANOVA) was used when two independent variables are involved (Figure 2). Analysis of covariance (ANCOVA) was performed using the R version 2.7.2 statistics package for the glucose tolerance tests (The R Project for Statistical Computing, a free online statistics package). An asterisk is indicated in all figures where statistical significance of p<0.05 was achieved.

## Results

### ZEB1 mRNA Expression Increases Concomitantly with Weight in WT Female Mice

Based on genome-wide association studies that mapped a gene linked to obesity in the region of chromosome 10 where *TCF8* is located (10p11.2) [9], [11]–[13] and on the roles of its resultant protein, ZEB1, in mesenchymal tissue differentiation [15]–[19], we speculated that ZEB1 contributes to the transcriptional regulatory cascades that modulate adipogenesis and/or lipogenesis. As a pilot study to address this question, the expression of ZEB1 was initially monitored in WT female mice. While cyclical fluctuations in circulating estrogen and progesterone are often considered an undesirable complication associated with using females, we reasoned that this was unlikely to be a significant problem because of the duration of our experiments (2–18 months) and that these hormones might even prove beneficial. As *TCF8* is induced about 10-fold by either estrogen or progesterone [24], [26], we predicted that more dramatic effects might be expected in female mice than in the males, which should have much lower levels of ZEB1. This issue is of particular importance for the subsequent experiments in this paper, which use mice that still retain one allele of *TCF8* because null mice are perinatal lethal [25]. Although haploinsufficiency of ZEB1 in humans and mice is sufficient to cause posterior polymorphous corneal dystrophy [41]–[43], no other significant phenotypes have been reported; so, it was not clear whether metabolic consequences could be detected in the heterozygotes. Thus, the prediction was that any effects of ZEB1 haploinsufficiency would be more apparent in females. Also, because the C57BL/6 males gain weight very rapidly and are obese by 3 months of age, we reasoned that the effects of ZEB1 might be obscured in them.

To determine whether ZEB1 levels vary with adiposity, ZEB1 mRNA expression was measured in the parametrial fat of WT female mice fed a RCD (regular chow diet) *ad libitum* or a calorie-restricted diet (75% of the calories consumed by the *ad libitum* group) from 2.5 to 18.3 months of age (Figure 1). The only difference in the diets was a reduction in the amount of carbohydrate in the restricted diet. As expected, the body weights of the *ad libitum* group almost doubled by 10 months of age, then plateaued for the duration of the experiment (Figure 1A). In contrast, the calorie-restricted mice exhibited no significant change in body weight. Real time PCR (qPCR) was done on parametrial tissues harvested from mice at 3.5, 6.3, 9.3, 13.8, and 18.3 months of age to measure ZEB1 and β-actin mRNA levels (Figure 1B). ZEB1 expression was the same with both dietary regimens at 3.5 months of age, but it increased dramatically with body weight in the *ad libitum* group and was significantly higher than those mice fed a calorie restricted diet by 9.3 months of age, although the trend was there by 6.3 months of age (Figure 1B). The same results were observed when the data were also corrected for body weight (data not shown). Similarly, ZEB1 protein levels also increased with body weight (Figure 1C), showing concordance between ZEB1 mRNA and protein expression and suggesting that ZEB1 regulates target genes in adipose tissue. Thus, an increase in the total body mass correlated with an increase in ZEB1 mRNA expression in parametrial fat, implying that ZEB1 has a specific role in adipose tissue.

### Female Mice Missing One TCF8 Allele Gain Excess Weight More Rapidly on a High-Fat Diet Than Do WT Controls

**Figure 3:**
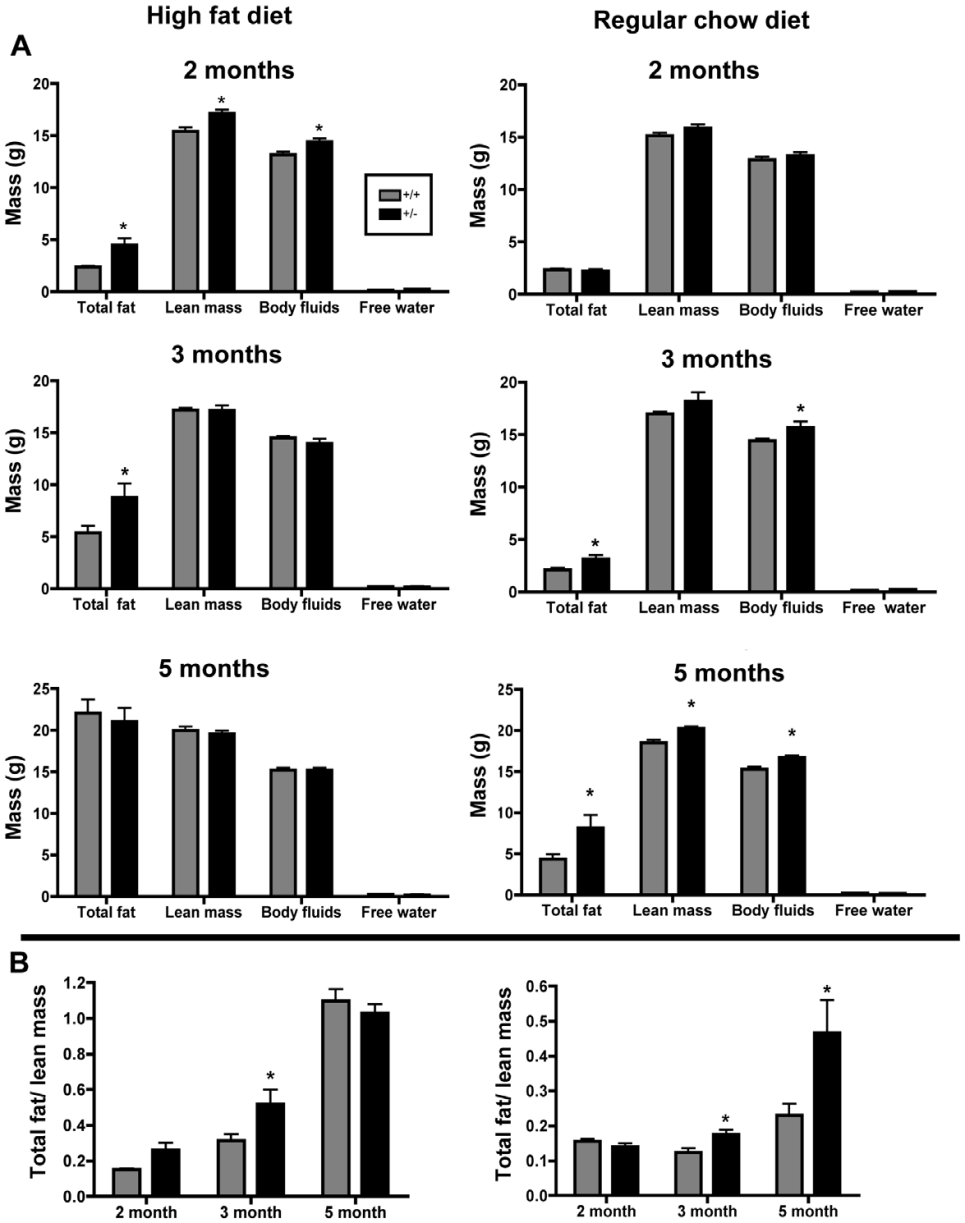
Female *TCF8*+/− mice fed a high-fat or regular chow diet have increased adipose mass early in fat acquisition. Echo MRI analysis of (**A**) whole body composition at 2, 3, and 5 months of age and (**B**) the fat/lean mass ratio of *TCF8+/−* (black bars) or WT (gray bars) of mice fed a high fat diet (left panel) or regular chow diet (right panel). Note the difference in the y-axes for (**B**). n = 4–8 mice per group. Only 4 mice were available for the 2 month-old group on the high fat diet.

To directly determine whether ZEB1 affects adipose accumulation, female mice lacking one *TCF8* allele (*TCF8*+/−) as determined by PCR (Figure 2C) and western immunoblot analysis (Figure 2D) were placed on a high fat diet (HFD, 60% of the calories from fat) or a RCD at weaning (21 days of age). *TCF8*+/- mice fed a HFD had significantly increased body mass by 1.3 months of age (Figure 2A). This increase in weight was maintained until 2.3 months of age, when the rate of weight gain started to decrease, and there was no difference in body weight between age-matched groups for the remainder of the experiment. We suspect that this is because sufficient fat mass has accumulated by 3 months to overcome any modulatory effects of ZEB1 (see Figure 3). These data indicate that ZEB1, directly or indirectly, reduces the early increase in body mass, during the time when the mice would be considered adolescent, when on a high fat diet.

On normal chow, the mice take longer to accumulate sufficient body weight to be able to detect significant differences between the genotypes (Figure 2B), and, as expected, they do not gain as much total weight as those on a HFD (the y-axes differ between Figure 2A and 2B). *TCF8+/−* mice maintained the same weight as WT controls on regular chow until 3.5 months of age, after which the ZEB1 heterozygous mice became significantly heavier. This trend continued until the termination of the study at 4.5 months of age. Note that the effects of ZEB1 haploinsufficiency with either diet occurred during a limited period or weight gain, when total body weights were increasing from about 20 to 27 grams and was thus not related to age of the animals as much as to actual body weight. The mechanistic reason for this restricted interval when ZEB1 haploinsufficiency had a measurable consequence is unknown. However, it is likely that once the mice on the high fat diet reach about 27 grams that both genotypes had sufficient fat to overcome the modulatory effects of ZEB1. Similarly, for mice fed a regular diet, too little fat was present below weights of ∼20 grams for ZEB1 to have a modulatory effect. Nonetheless, these experiments indicate that there is a substantial interval where total body mass was higher in the ZEB1 heterozygotes than in age-matched WT controls.

### Increased Body Weight in TCF8+/− Female Mice Is Due to an Increase in Adipose Mass

To ascertain whether the increase in body weight exhibited by the *TCF8*+/− mice can be ascribed to increased adipose mass or to other contributors such as lean muscle mass, Echo MRI was used to measure total fat mass, lean mass, free water, and total body fluids on mice fed either the HFD or RCD (Figure 3). There was no difference in free water between *TCF8*+/− mice and WT controls at any age on either diet. Interestingly, on a HFD, *TCF8*+/− mice exhibited increased total fat mass by 2 months of age, which was maintained at 3 months (Figure 3A, left panel). By 5 months, both groups had a high fat mass, and no difference was observed with genotype. However, an increase in lean mass was also observed in *TCF8*+/− mice at 2 months of age, suggesting that these young mice are heavier overall and that both lean mass and fat mass contributed to the difference in total body fluids and overall body weight at this early age. These mice were not significantly longer that the WT mice as determined by measurement from nose to tail (data not shown). To determine whether the increase in body weight at 2 months is primarily a result of increased adipose tissue or increased lean mass, the ratio of fat mass to lean mass was calculated (Figure 3B, lowest left panel). Although there was a trend for a higher total fat to lean mass ratio in the mice fed a high fat diet at 2 months, only 4 mice were available for that time point and statistical significance was not achieved. In contrast, the increased body weights of the heterozygous mice at 3 months can be ascribed entirely to increased fat mass. These data as a whole indicate that the increase in body weight was due primarily to an increase in fat mass, not just to an overall increase in the size of the mice or lean muscle mass.

Whole body composition by Echo MRI was also done on *TCF8+/−* and WT female mice fed regular chow (Figure 3A, right panel). In agreement with the body weight data (Figure 2B), *TCF8*+/− mice had no difference in adipose mass at 2 months of age (Figure 3A, right panel). However, at both 3 and 5 months of age the *TCF8*+/− mice had increased total body fat compared to WT mice. Similarly, the ratio of fat mass to lean mass also increased in *TCF8+/−* mice at 3 and 5 months of age (Figure 3B, lowest right panel). Taken together, these data suggest that ZEB-1 specifically reduces adipose mass in female mice regardless of the diet consumed.

### TCF8+/− Mice Do Not Store the Increased Fat Mass Preferentially in the Parametrial Fat Pads

**Figure 4:**
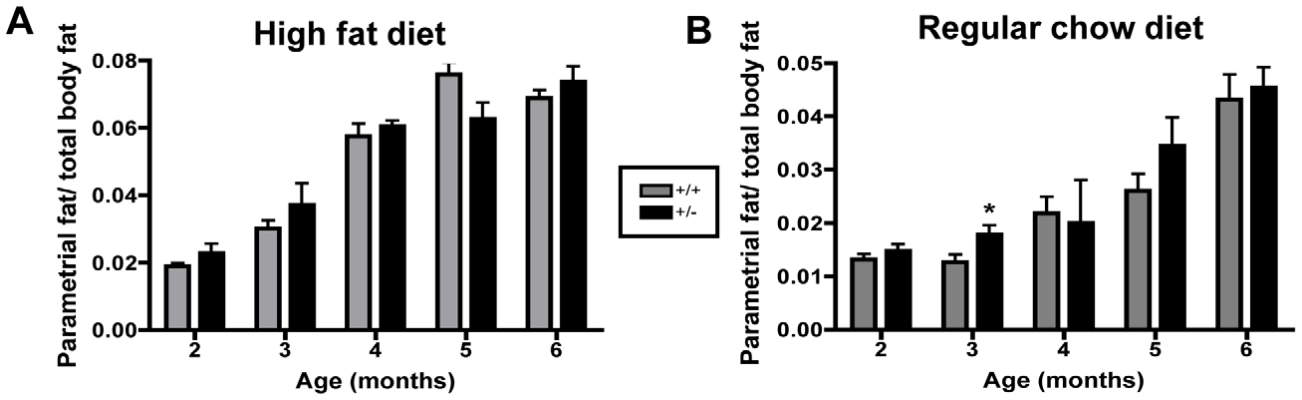
The mass of parametrial fat pads does not differ with genotype. The mass of parametrial fat pads for *TCF8+/−* (black bars) and WT controls (gray bars) ages 2–6 months for mice fed (**A**) a high fat diet or (**B**) regular chow was determined by weighing. Note differences in the y-axes. Data are represented as a ratio of parametrial fat weight to total body weight. n = 5–12.

Differential gene expression occurs in the various adipose depots [44], and ZEB1 expression is higher in visceral fat than in subcutaneous fat [21]. ZEB1 is also highly expressed in the human uterus [26], [27] and is estrogen-responsive in multiple tissues [24], [26], [27], [31]. For these reasons, we hypothesized that ZEB1 may have a greater impact on the parametrial (gonadal) fat pads than on the other adipose depots. Parametrial fat pads were harvested monthly (2–6 months of age) from *TCF8*+/− female mice and WT controls fed either a HFD or RCD (Figure 4). No significant differences were observed with regard to genotype or diet except at 3 months, where the *TCF8+/−* mice fed regular chow had significantly higher parametrial fat pad weights compared to WT controls. However, these data *in toto* suggest that ZEB1 does not act locally in gonadal depots to affect fat deposition but instead exerts its effects more globally.

### Increased Body Fat in TCF8+/− Mice Is Not Due to Increased Food Consumption

**Figure 5:**
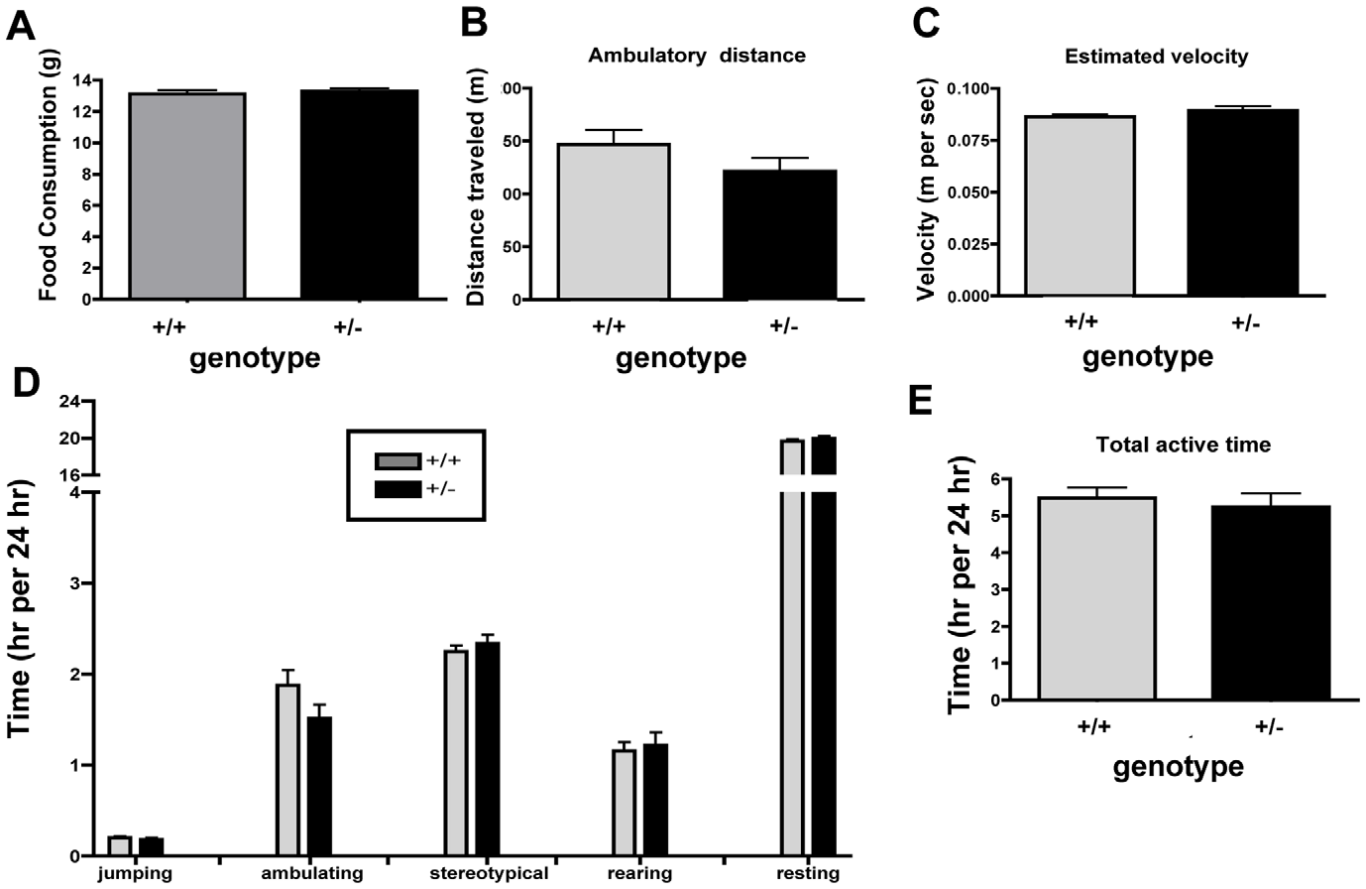
Increased fat accumulation is not the result of increased food consumption or decreased physical activity. (**A**) Food intake was measured for 72 hours in 2.5-month-old *TCF8+/−* and WT mice fed regular chow. n = 10 mice per group. (**B**) The total distance the mice moved. (**C**) The estimated velocity at which the mice moved. (**D**) The duration of each activity or time spent resting. (**E**) Total duration the mice spent performing any activity. *TCF8+/−* mice are depicted by black bars and WT controls by gray bars. n =  15–17 mice per group for **B–E**. No significance differences were found for time spent on any activity, resting, total activity, estimated velocity, or number of times an activity was performed.

One explanation for the increased adipose tissue accumulation by the *TCF8*+/− mice on both the high and low fat diets is that they might consume more calories. To assess this, the weight of regular chow consumed by 2.5 month old *TCF8+/−* and WT female mice over a period of three light and dark cycles was measured (Figure 5A). No significant differences in food intake were observed in any of the three repetitions. The body weights of 2.5-month-old mice fed regular chow are not statistically different between the genotypes (Figure 2B and data not shown), so metabolic changes as a result of increased body weight do not compromise this assessment. These data indicate that ZEB1 has no impact on caloric consumption.

### Increased Adiposity of Female TCF8+/− Mice Is Not Due to Decreased Physical Activity

As the *TCF8*+/− mice do not consume more calories, another explanation for their enhanced adiposity is that they might be more sedentary. Evidence in *Drosophila* suggests that total loss of the ZEB1 ortholog causes impaired development of neuromuscular junctions [45], [46]. This raises the possibility that the *TCF8*+/− mice may have minor muscle or motor neuron developmental deficiencies, which could restrict their mobility and thus reduce caloric expenditure. In addition, female mice primarily alter energy expenditure rather than energy intake to regulate body weight [33], so physical activity was extensively characterized. Three-month-old *TCF8+/−* and WT mice fed regular chow were monitored in an activity chamber for 24 hrs (Figures 5B–E). Laser breaks recorded movement both horizontally and vertically. Activity was quantified for the actual ambulatory distance (Figure 5B) and for the duration or time spent performing an activity (Figure 5D). The velocity at which ambulation took place was estimated by calculating m/sec (Figure 5C). No differences were observed in any type of activity or overall time spent being active (Figure 5E, p = 0.3395). Thus, these data imply that significant metabolic differences must exist between the two genotypes that contribute to differences in fat accumulation.

### TCF8+/− Mice Have a Window of Impaired Glucose Uptake

**Figure 6:**
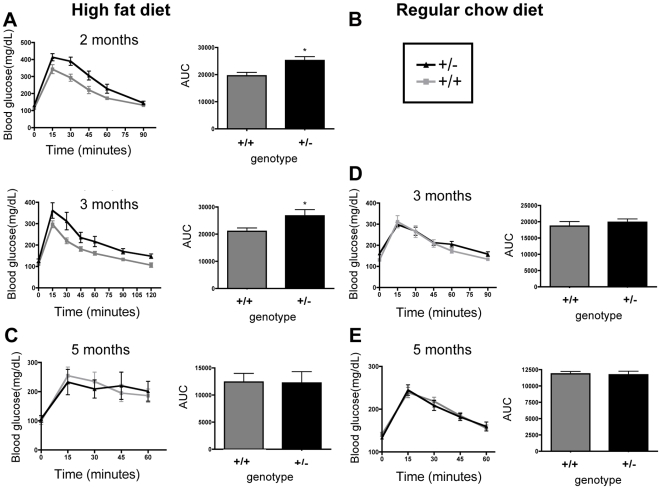
Female *TCF8+/−* mice exhibit impaired glucose uptake early in fat acquisition. Mice were fed a high fat diet (**A–C**) or regular chow (**D, E**) until they were 2, 3, or 5 months of age. Blood glucose was measured at the indicated times following injection of glucose at 2 mg/kg (**A, B, E**), 0.5 mg/kg (**C**), or 3 mg/kg (**D**). Area under the curve (AUC) was calculated and graphed as histograms. When Student's *t-*test was used to analyze the AUC, the *TCF8+/−* mice were significantly different from WT in their ability to manage blood glucose levels as indicated by the asterisks. ANCOVA was performed for **A** and **B** with body fat as a co-variate for the differences in glucose tolerance between genotype to assess whether genotype contributed independently of fat mass. For **A**: genotype p = 0.006, body fat p = not significant, for **B**: genotype: p =  not significant, body fat p =  not significant. *TCF8 +/−* (black line) or WT (gray line), n = 5–8 mice per group.

Obesity has many metabolic consequences. To investigate whether weight differences due to the loss of one copy of *TCF8* are sufficient to disrupt metabolic homeostasis, glucose tolerance tests were performed on *TCF8+/−* and WT controls fed either a HFD or RCD (Figure 6). Mice were injected with a bolus of glucose that ranged from 0.5–3 mg/kg depending upon their body weight. At 2 and 3 months of age (Figures 6A and B, respectively), *TCF8+/−* mice fed a HFD did not process glucose as well as WT controls. Analysis of co-variance (ANCOVA) using body fat as a covariate revealed that the *TCF8+/−* genotype contributed to the difference at 3 months of age, not just the fat mass. However, ANCOVA could not determine whether genotype contributed to the statistical difference at 2 months of age. As expected, there was no difference in glucose utilization at 5 months of age (Figure 6C), presumably because both groups had excess fat mass by that time (Figure 3). On a RCD, there was no difference in glucose uptake between *TCF8*+/− mice and WT controls at either 3 or 5 months of age (Figures 6D and E). While there is significantly more adipose tissue in the heterozygous mice (Figure 3), the actual mass appears to not be enough to confer decreased glucose uptake. These data demonstrate, as anticipated, that glucose processing is impaired in overweight mice. Of more interest, ZEB1 haploinsufficiency independently reduces glucose uptake during part of the time when the mice are gaining excessive weight while on a HFD.

### ZEB1 Expression Increases During Adipogenesis

**Figure 7:**
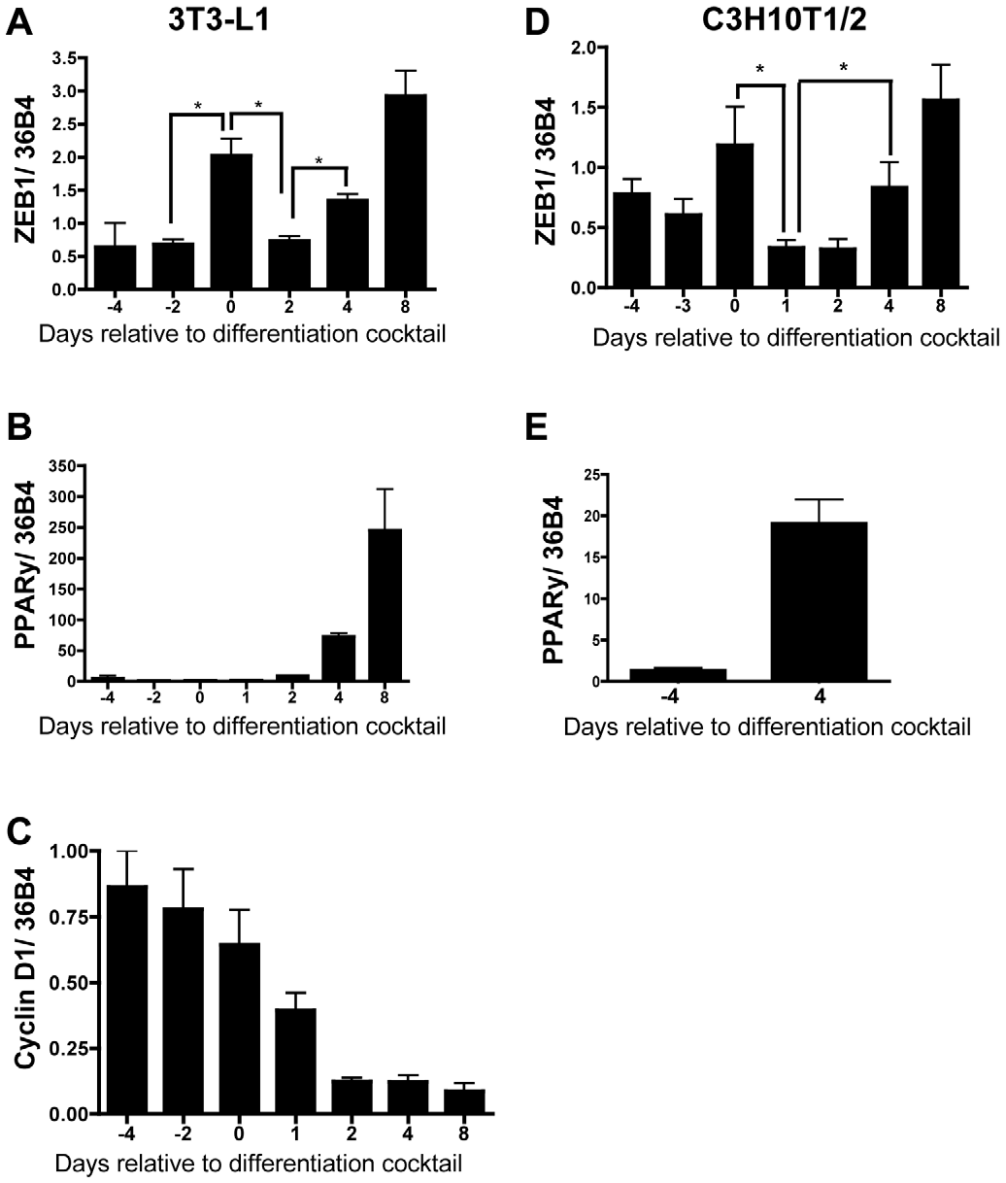
ZEB1 expression changes similarly in two models of adipogenesis in cell culture. 3T3-L1 cells (left panels) were differentiated from pre-adipocytes into mature adipocytes. Cells reached confluency at Day -2 and were treated with a differentiation cocktail on Day 0. RNA was harvested in triplicate on the days indicated, and mRNA expression were measured by qPCR for ZEB1 (**A**), PPARγ (**B**), and Cyclin D1 (**C**). C3H10T1/2 pluripotent mesenchymal stem cells (right panels) were committed to the pre-adipocyte lineage by treatment with BMP-4 at Day -4, when they were 75% confluent. Cells reached confluency at Day -2 and were treated with differentiation cocktail on Day 0. RNA was harvested in triplicate at the days indicated and subjected to qPCR for ZEB1 (**D**) and PPARγ (**E**). All mRNA levels were normalized to ribosomal protein 36B4. These experiments are representative of differentiations done 5 and 6 times, respectively.

ZEB1 is involved in the development of several tissues, opposing the differentiation of some mesenchymal tissues [15], [17], [47]–[50] and promoting the differentiation of others including T-cells [25] and smooth muscle cells [48], [51], [52]. To explore the role, if any, that ZEB1 has in adipose tissue development, endogenous ZEB1 mRNA levels were measured throughout differentiation of NIH3T3-L1 pre-adipocytes into mature adipocytes. The cells were induced to differentiate as described in Methods, and ZEB1 mRNA expression was measured using qPCR on the indicated days relative to treatment with differentiation cocktail (Figure 7A). ZEB1 expression shows about a 4-fold increase in ZEB1 mRNA levels when the cells go from proliferating (Days -4 and -3) to confluency (Day 0). By Day 2, ZEB1 mRNA levels return to that observed in pre-adipocytes and then increase to maximal levels (about 5-fold) by four days after treatment. The changing ZEB1 mRNA levels suggest that it may have a role in the early and late stages of adipocyte development. This is in agreement with our observations in mice where ZEB1 increases in response to increased adipose accumulation (Figure 1). PPARγ and cyclin D mRNAs were also measured to serve as positive controls for adipocyte development (Figures 7B and C). The expression of both mRNAs was consistent with proper adipocyte differentiation.

Because 3T3-L1 cells are already at the pre-adipocyte stage, they can not be used to assess whether ZEB1 affects commitment to the adipocyte lineage. To determine whether ZEB1 expression is altered prior to commitment to the adipocyte lineage, the C3H10T1/2 pluripotent mesenchymal cell line was used. The cells were treated with BMP-4 at Day -4 to commit them to the pre-adipocyte lineage [40], [53]. Once they were pre-adipocytes and reached confluency, they were triggered at Day 0 to differentiate into adipocytes with the same differentiation cocktail as was used for the 3T3-L1 cells. Endogenous ZEB1 mRNA expression was measured throughout commitment and differentiation at the indicated time points (Figure 7D and E). A similar trend was observed with the C3H10T1/2 cells as was seen with the 3T3-L1 cells. ZEB1 mRNA levels did not change upon commitment of the cells to the adipocyte lineage (Day -3), but it did increase at confluency (Day 0), decrease with the initiation of differentiation (Day 1), and then increase with lipid accumulation (Day 4). PPARγ was measured (Figure 7E) and cells were stained for oil red O (data not shown) to confirm a high level of differentiation. These data suggest that ZEB1 either does not regulate commitment to the adipocyte lineage or that sufficient ZEB1 is already present in the mesenchymal precursor cells.

## Discussion

Based on genome wide association studies that link obesity to the region on chromosome 10 that encompasses the *TCF8* gene [9], [11]–[13] and on the ability of ZEB1 to impact the differentiation of other mesenchymal cell lineages [15]–[19], we hypothesized that ZEB1 opposes obesity. Using *TCF8+/−* mice, we demonstrate herein that ZEB1 haploinsufficiency elicited excessive adipose tissue accumulation in female mice early in fat acquisition (Figures 2 and 3). This increased adiposity occurred on both regular chow and high fat diets, although significant differences in body weight were delayed by one month on the regular diet. These data suggest that ZEB1 is an important modulator of adipose tissue mass, not just under circumstances of high caloric pressure but also with a normal diet. However, there is one important caveat to this interpretation. Knockout of *TCF8* was accomplished by insertion of a 3.8 Kb DNA fragment containing the β-galactosidase gene into the first exon. Thus, this insertion into the *TCF8* locus could be causing the effects on adipose accumulation. While this cannot be ruled out, the observations that endogenous ZEB1 expression increases in the adipose tissue of WT mice with increased fat mass (Figure 1B) and that ZEB1 fluctuates similarly in two WT cell lines during adipogenesis (Figure 7) argues against an off-target effect of this insertion.

In order to begin to delineate the potential mechanisms by which ZEB1 represses obesity, food intake and activity were measured. No differences were observed in food consumption between *TCF8*+/− and WT mice or in the total amount or duration of physical activity (Figure 5), which indicates that ZEB1 is affecting something other than energy intake or expenditure. This raises the question of whether ZEB1 is acting directly in adipose tissue and/or in other tissues. Certainly most of the genes associated with monogenetic or syndromic obesity described thus far are expressed in the brain [54]. However, the large increase in ZEB1 expression in parametrial fat pads that occurs as the mice gain weight (Figure 1B) implies at least some role for ZEB1 in adipose tissue. Furthermore, the parallel fluctuations in ZEB1 expression observed during adipogenesis in two cell lines (Figure 7) suggest that ZEB1 is acting, at least in part, locally within the adipocyte.

The effects of ZEB1 haploinsufficiency were restricted, at least in this experimental paradigm, to a period of a few weeks that correspond to when the mice are initially gaining fat and are about 20 – 27 g in size. When the mice are smaller, ZEB1 haploinsufficiency does not affect adiposity, perhaps because they are growing rapidly and little fat is accumulating. When the mice have matured and have gained almost 4 g of fat tissue, then significant differences in fat mass exist between the *TCF8+/−* and WT mice (Figures 2A and B). The fact that ZEB1 expression increases dramatically in adipose tissue with fat accumulation (Figure 1B) complicates these experiments as there were no differences in ZEB1 levels between the genotypes at 4 or 5 months of age (data not shown). This latter point raises a paradox that exists with these data, the observation that ZEB1 mRNA increases in mice as they gain adipose mass, yet ZEB1 haploinsufficiency leads to increased fat accumulation (Figure 3). High expression of ZEB1 has also been reported in stem cells stimulated to differentiate into adipocytes *in vitro* [2] and in obese women compared to those of normal weight [21]. Thus, ZEB1 opposes fat accumulation yet is highly expressed in fat. One plausible explanation is that ZEB1 increases in order to repress additional fat accumulation. It is also possible that ZEB1 has multiple roles in modulating adipogenesis and adiposity. Many of these questions will need to be resolved using adipose tissue-specific *TCF8* knockouts.

Another unexpected result is the increase in lean mass as well as fat mass in *TCF8+/−* mice at 2 months of age on a HFD. This did not occur on the RCD at either 2 or 3 months of age or on the HFD at 3 months of age. While this could be an experimental anomaly, this seems unlikely as the mice were all from the same breeder parents, were age-matched, and were housed in the same room. One possibility relates to the observations that ZEB1 opposes skeletal muscle differentiation in *Drosophila* [55] and in cell culture [16]. Diminished amounts of ZEB1 could lead to increased muscle differentiation and thus lean mass in a context where the mice are rapidly gaining in body mass, i.e., a HFD. However, the *TCF8* null mouse does not exhibit enhanced muscle mass [15]. Instead, its overall size is diminished with no obvious histological differences in the muscle system or in the expression of key myogenic genes. In contrast, heterozygous mice appear morphologically normal [15]. Thus, the increase in lean muscle mass in our mice likely relates in some way to the rapid increase in body weight and fat mass when they are on a HFD.

Insufficient data are available to assess whether diminished ZEB1 mass or function affects adiposity in humans. Two studies demonstrated that one form of posterior polymorphous corneal dystrophy (PPCD) is caused by heterozygous mutations in *TCF8* [41], [42]. Of the 9 patients in those studies who have mutations in *TCF8*, none were reported to be obese or overweight. Thus, it is possible that ZEB1 haploinsufficiency has no affect on fat accumulation in humans. Alternatively, as these mutations are mostly in Exon 7 (of 9), some functions of ZEB1 may be retained. Certainly the four N-terminal zinc fingers should be intact and able to bind DNA in all of these patients. In addition, as all these patients have visual problems and most have other abnormalities, it is possible that poor health contributes to their lean phenotype. Additional studies will obviously be required to determine whether ZEB1 affects adiposity in humans.

Another unanswered question is whether ZEB1 deficiency contributes to fat accumulation in males as well as females. Because ZEB1 is induced by estrogen [24], [26], [27], [31] and because estrogen influences a number of metabolic events that affect adiposity and glucose homeostasis [32], [33], we decided to use female mice for these initial studies. Even though these mice are cycling through the estrous cycle and thus have fluctuating estrogen and progesterone levels, we predicted that ZEB1 levels would be higher in females than in males because these sex steroid hormones induce *TCF8* in females [24], [26], [56]. However, it is possible that significant effects of ZEB1 haploinsufficiency on some parameters were overlooked in our study because of the relatively small numbers of mice used per treatment group and because their estrous cycles were not synchronized. Furthermore, because progesterone and testosterone may oppose estrogen's effects [57], more dramatic consequences of genotype may have been seen in ovariectomized animals treated with estrogen. The fact that significant effects of ZEB1 haploinsufficiency on fat accumulation (Figure 3) and glucose uptake (Figure 6) were observed under these conditions attests to the importance of ZEB1 in modulating energy homeostasis. Nonetheless, we anticipate that ZEB1 mediates at least some of the effects of estrogen in attenuating fat accumulation. Thus, even in these reproductively intact mice, estrogen is unable to signal properly because of reduced ZEB1 levels. Experiments are in progress with male mice and with anti-estrogen treated female mice to assess whether ZEB1 mediates estrogen's effects on fat metabolism, whether it acts independently of estrogen, or both.

Even though ZEB1 insufficiency contributes to fat accumulation only during the time when fat mass is modest, this is a critically important time. Considerable evidence indicates that once fat is accrued in humans, especially in adolescents, it is difficult to reverse that and most individuals remain overweight or obese for life [58], [59]. Thus, the observation that ZEB1 modulates adiposity during this early period of fat accumulation may have important implications for preventative measures. This is particularly true for females as adipose tissue-specific estrogen receptor agonists could be developed to locally modulate ZEB1 levels.

Transcriptional cascades regulate the differentiation of mesenchymal stem cells into mature adipocytes [38], [60]. While the major players have already been identified, modulators such as ZEB1 are still being elucidated. As most genetic causes of obesity are polygenic [6], a complete understanding of the proteins involved will be necessary to determine when a cell commits to the adipocyte lineage, when it differentiates into an adipocyte, and how lipids are stored within adipocytes. The data herein demonstrate for the first time that a diminished amount of the ZEB1 transcription factor can lead to increased adiposity during early weight gain, at least in females. They also substantiate previously published genetic association studies implicating the *TCF8* locus as one of the loci on chromosome 10 linked to childhood obesity [9], [11]–[13]. The goal now is to determine how ZEB1 fits into the transcriptional pathways that modulate fat metabolism.
